# Supplementary material for: A new sponge-associated starfish, Astrolirus patricki sp. nov. (Asteroidea: Brisingida: Brisingidae), from the northwestern Pacific seamounts
Source: PeerJ. 2020 May 27;8:e9071. doi: 10.7717/peerj.9071 (PMC7261123; doi:10.7717/peerj.9071)
Supplement: Table S1 [file peerj-08-9071-s001.docx]

**Table S1. GenBank accession numbers of taxa used in phylogenetic analyses.**

| Species name | Specimen voucher | COI | 16S | H3 | 12S | 18S |
| --- | --- | --- | --- | --- | --- | --- |
| *Astrolirus patricki* ***sp. nov.*** | RSIOAS044 | MN885905 | MN879474 | MN963770 | MN879483 | MN879541 |
| *Astrolirus patricki* ***sp. nov.*** | RSIOAS028 | MN885903 | MN879472 | MN963768 | MN879481 | MN879539 |
| *Astrolirus patricki* ***sp. nov.*** | RSIOAS003 | MN885899 | MN879468 | - | MN879477 | - |
| *Astrolirus patricki* ***sp. nov.*** | RSIOAS052 | MN885906 | MN879475 | MN963771 | MN879484 | MN879542 |
| *Astrolirus patricki* ***sp. nov.*** | MBM286625 | MN885907 | MN879476 | MN963772 | MN879485 | MN879543 |
| *Brisinga sp.*1 | RSIOAS007 | MN885900 | MN879469 | MN963767 | MN879478 | MN879536 |
| *Brisinga sp.*2 | RSIOAS023 | MN885902 | MN879471 | - | MN879480 | MN879538 |
| *Hymenodiscus* cf. *fragilis* | RSIOAS009 | MN885901 | MN879470 | - | MN879479 | MN879537 |
| *Freyella* cf. *attenuata* | RSIOAS037 | MN885904 | MN879473 | MN963769 | MN879482 | MN879540 |
| *Freyastera delicata* | RSIOAS022 | MK695796 | MK696054 | MT127565 | MN028399 | MN028404 |
| *Freyastera basketa* | RSIOAS038 | MK695800 | MK695794 | MK695804 | MN028402 | MN028405 |
| *Freyastera basketa* | RSIOAS039 | MK695801 | MK695795 | MK695805 | MN028403 | MN028406 |
| *Freyastera basketa* | RSIOAS006 | MK695798 | MK695792 | MK695802 | MN028400 | - |
| *Freyastera basketa* | RSIOAS008 | MK695799 | MK695793 | MK695803 | MN028401 | - |
| *Freyastera* sp. Yap | RSIOAS041 | MK695797 | MK696055 | - | - | - |
| *Freyastera benthophila* | isolate CLM-212 | - | EU722993 | EU707770 | - | - |
| *Freyastera benthophila* | - | NC039982 | NC039982 | - | NC039982 | - |
| *Freyastera* cf. *benthophila* NHM-413 | NHMUK: MCF 185546349 | KU519550 | KU519518 | - | - | KU519535 |
| *Freyastera* cf. *benthophila* NHM-421 | NHMUK: MCF 185546363 | KU519551 |  | - | - | - |
| *Freyella* sp. 1 RDW-2008 | Ech177 | EU869921 |  | - | - | - |
| *Freyella* sp. CLM-2011-1 | CASIZ 163025 | - | EF624406 | EU707648 | - | - |
| *Freyella* sp. CLM-2008 | CASIZ 163058 | - | EU072958 | - | - | - |
| *Freyellaster* *fecundus* | RBCM EC00154 | HM400321 | - | - | - | - |
| *Hymenodiscus pannychia* | RBCM EC00027 | HM542932 | HM543145 | - | - | - |
| *Hymenodiscus* sp. USNM E47614 | USNM E47614 | - | EU722922 | EU707659 | - | - |
| *Hymenodiscus* sp. MNHNP EcAh 6036 | MNHNP EcAh 6036 | - | EU722977 | EU707747 | - | - |
| *Astrostephane moluccana* | NIWA 33311 | - | GQ288556 | - | - | - |
| *Odinella nutrix* | CAS 163023 | DQ077927 | AY706145 | - | - | AY935550 |
| *Brisingaster robillardi* | -/MNHNP EcAh 4958/- | AF217392 | EU722978 | - | - | AF088802 |
| *Novodinia novaezelandiae* | NIWA 31992 | - | EU722991 | EU707772 | - | - |
| *Novodinia antillensis* | CASIZ-USNM s.n. | - | EF624412 | - | - | - |
| *Stichaster striatus* | -/isolate N39 | JX129954 | EF624422 | EU707677 | - | - |
| *Cosmasterias lurida* | isolate N42 | - | EF624402 | EU707679 | - | - |
